# Saliva-acquired pellicle inspired multifunctional gargle with wet adhesion, photodynamic antimicrobial, and In situ remineralization properties for dental caries prevention

**DOI:** 10.1016/j.bioactmat.2025.01.008

**Published:** 2025-01-23

**Authors:** Jiayi Shi, Xuekai Qi, Ying Ran, Qiang Zhou, Yiqin Ding, Lujian Li, Youyun Zeng, Dongchao Qiu, Zhibin Cai, Xiaojun Cai, Yihuai Pan

**Affiliations:** a School and Hospital of Stomatology, Wenzhou Medical University, Wenzhou, 325027, China; b Department of Endodontics, School and Hospital of Stomatology, Wenzhou Medical University, Wenzhou, China; c The Third Affiliated Hospital of Wenzhou Medical University (Ruian People's Hospital), Wenzhou, 325016, China

**Keywords:** Saliva-acquired pellicle, Wet adhesion, Tooth remineralization, Antimicrobial photodynamic therapy, Caries prevention, Interrupting dental caries

## Abstract

Dental caries is primarily caused by cariogenic bacteria metabolizing carbohydrates to produce acidic substances that erode the dental hard tissues. Traditional remineralization treatments often have limited efficacy due to their lack of antibacterial activity. According to the Interrupting Dental Caries (IDC) theory, ideal caries-preventive materials should possess both antibacterial and remineralizing properties. Furthermore, effective adhesion to dental surfaces is crucial. Inspired by the wet adhesion properties of the salivary acquired pellicle, we developed a multifunctional gargle named Ce6@PDN-SAP (CP-SAP). This formulation employed peptide dendrimer nanogels (PDN) as a carrier for the photosensitizer Ce6, further functionalized with saliva-acquired peptide (SAP) to confer wet adhesion properties. CP-SAP rapidly adhered to the dental surface and remained effective for extended periods. Upon laser irradiation, Ce6 generated reactive oxygen species (ROS), disrupting bacterial outer membrane integrity, causing protein leakage, and reducing ATP levels, thereby achieving potent antibacterial effects. Following this, PDN and SAP acted as nucleation templates to promote in situ remineralization of demineralized dental hard tissues. In vivo studies using rat models demonstrated that CP-SAP provided significantly superior caries-preventive effects compared to chlorhexidine gargle. In conclusion, CP-SAP, as an innovative approach grounded in the IDC theory, holds great promise for the prevention and treatment of dental caries.

## Highlights

• We developed a multifunctional gargle solution (CP-SAP), based on a peptide dendrimer nanogel (PDN) modified with saliva-acquired peptide (SAP), serving as an innovative drug delivery carrier with wet adhesion and in situ remineralization properties, capable of efficiently delivering various drugs, including photosensitizers.
• CP-SAP's excellent wet adhesion allows it to adhere to the dental surfaces, providing long-lasting therapeutic effects.
• CP-SAP demonstrates strong photodynamic antimicrobial efficacy, rapidly eliminating cariogenic bacteria and inhibiting biofilm formation.
• PDN and SAP also act as nucleation templates, effectively adsorbing calcium and phosphate ions from saliva to promote rapid remineralization of demineralized dental hard tissues.
• In vitro and in vivo studies show that CP-SAP's antibacterial and remineralization effects are comparable to, or superior to, chlorhexidine gargle and fluoride varnish, highlighting its potential in dental caries prevention and treatment.

## Graphical abstract

**Figure:**
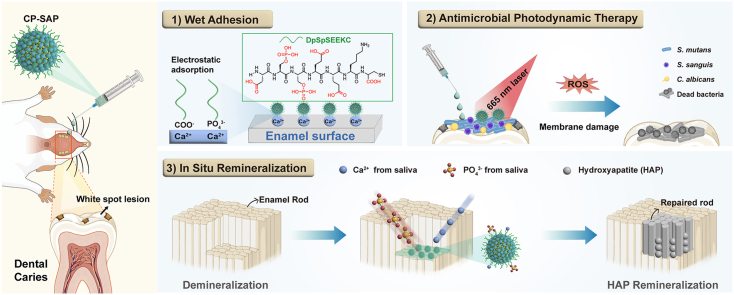

## 1 Introduction

Dental caries pose a significant global health challenge due to their high prevalence [1] and treatment failure rates [2]. These conditions arise when oral bacteria metabolize carbohydrates, producing acids that demineralize dental hard tissues [3]. In the early stages, non-cavitated white spot lesions appear on the tooth surface, signaling bacterial-mediated demineralization [4]. At this stage, treatment is more straightforward and simpler, as cavities have not yet formed [5]. Chlorhexidine (CHX) gargle and fluorides are widely used in clinical practice to prevent and treat early caries by inhibiting cariogenic bacteria metabolism or promoting remineralization [6,7]. However, their effectiveness is limited due to their singular mechanisms—either antimicrobial action or remineralization. The emergence of fluoride-resistant bacterial strains [8] and concerns about systemic issues related to excessive fluoride intake, such as dental and skeletal fluorosis, further complicate fluoride use [9]. Additionally, CHX is unsuitable for long-term use due to adverse effects like tooth staining and taste alteration [10]. To overcome the limitations of current treatments, Gu et al. developed stimuli-responsive graphdiyne-silver nanozymes for catalytic ion therapy in dental caries, emphasizing their antibacterial activity and enhanced remineralization effects [11]. Similarly, Dai et al. proposed a VMT/ACP/Dextran composite nanosheet system, combining antibacterial properties, dentin tubule remineralization, and acid buffering abilities [12]. Furthermore, Zhang et al. proposed an innovative strategy for caries prevention and treatment, termed “Interrupting Dental Caries (IDC)” which centers around the use of multifunctional materials to provide early intervention and treatment of caries. This approach integrates properties such as remineralization, antibacterial activity, and permeability, enabling a comprehensive and effective treatment across various stages of caries progression [13,14]. These studies underscore the urgent need to develop new multifunctional materials that can overcome the limitations of current treatments and offer safer, more effective solutions for the prevention and management of dental caries.

Antimicrobial photodynamic therapy (aPDT), an emerging non-invasive antimicrobial treatment, generates reactive oxygen species (ROS) under laser irradiation, to damage bacterial cell membranes and cell walls through photosensitizers [15,16]. With excellent antimicrobial effects and a low risk of inducing bacterial resistance [17,18], aPDT has shown considerable promise for dental caries prevention [19,20]. However, traditional photosensitizers face challenges such as limited water solubility [21], and in the dynamic oral microenvironment, they struggle to maintain adhesion to dental surfaces due to the washing effects of saliva. Moreover, the therapeutic potential of aPDT is limited when used alone. Therefore, to fully leverage the potential of aPDT in interrupting dental caries, it is crucial to develop a photosensitizer delivery system that combines wet adhesion and remineralization properties, which would significantly enhance its therapeutic efficacy.

Peptide dendrimer nanogels (PDN) are a promising class of biomedical materials [22,23], valued for their ability to deliver both hydrophilic and hydrophobic drugs [24,25]. Compared to traditional dendrimers, PDN, when crosslinked, retain the biodegradability of nanogels and exhibit superior biocompatibility and stability, essential for maintaining effective drug delivery within biological systems [26]. Additionally, the presence of abundant amino functional groups on the periphery of PDN enables facile functionalization [[27], [28], [29], [30]], a pivotal attribute that can be harnessed to confer wet adhesion properties upon PDN. The salivary acquired pellicle is a biological film composed of proteins and glycoproteins that forms in the oral cavity and can tightly adhere to the dental surface [31]. Within this film, statherin plays a central role [32], and the first six amino acid sequence at its N-terminus—DpSpSEEK, also known as the salivary acquired peptide (SAP) sequence—is the key structural domain that endows it with strong adhesive capabilities [33]. By functionalizing PDN with SAP, we can significantly improve their wet adhesion to dental surfaces. Furthermore, both PDN and SAP can act as nucleation templates to promote remineralization of demineralized dental hard tissue. PDN can stabilize phosphate ions in saliva through their terminal amino groups, while the phosphate and carboxyl groups on SAP can capture free calcium ions [34], synergistically facilitating the growth of hydroxyapatite (HAP) and promoting in-situ remineralization. Thus, PDN functionalized with SAP serve as an ideal delivery carrier for photosensitizers, capable of adhering to dental surfaces, exerting antibacterial photodynamic therapy effects, and promoting remineralization. This integrated approach addresses the challenges of poor water solubility and inadequate adhesion commonly associated with traditional photosensitizers in aPDT, while also overcoming the limitations of single functionality, offering a more effective and multifunctional solution for caries prevention.

**Scheme. 1:**
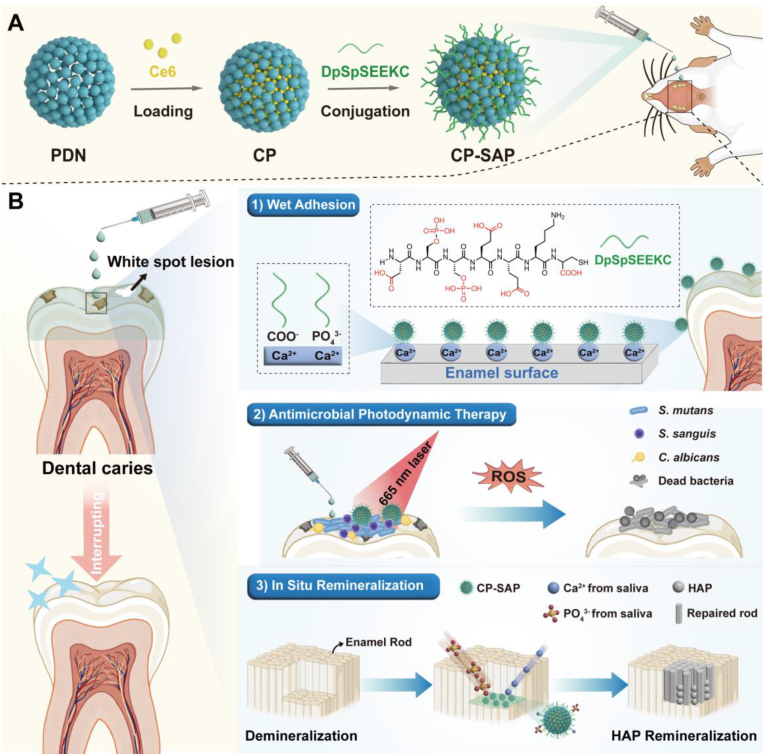
Schematic diagram illustrating the CP-SAP A) preparation process and B) mechanisms of wet adhesion, antimicrobial photodynamic therapy, and in situ remineralization.

In this study, we developed a multifunctional gargle (Scheme 1) by physically loading chlorin e6 (Ce6) into SAP-modified PDN (CP-SAP). This novel formulation leverages the advantages of SAP, PDN, and aPDT, with the following key attributes: 1) rapid adhesion and long-lasting retention on the dental surface; 2) effectively inhibits the growth of cariogenic bacteria and biofilm formation via Ce6-mediated aPDT; 3) ability of the SAP and PDN to act as nucleation templates and synergistically promote in situ remineralization of demineralized dental hard tissues. We carefully investigated its in-vitro wet adhesion, photodynamic antibacterial, and remineralization-promoting capabilities of CP-SAP. Additionally, its in-vivo efficacy was rigorously tested in rat caries models.

## 2 Materials and methods

### 2.1 Reagents

G2-Lys was synthesized in the laboratory [35]. Di (N-succinimidyl) 3,3′-Dithiodipropionate (DSP), Deuterium oxide and Cy5-NHS were obtained from Aladdin (Shanghai, China). DpSpSEEKC was purchased from Tanshtech Co., Ltd (Guangzhou, China). Sodium chloride, Chlorin E6, 3-Maleimidopropionic acid, and N-Hydroxy succinimide were provided by Macklin (Shanghai, China). 1-Ethyl-3-(3-dimethylaminopropyl) carbodiimide and Artificial saliva were obtained from Shanghai Yuanye Bio-Technology Co., Ltd (China). L929, HGF cells and HGFs cell culture medium were sourced from Otwo Biotech (China). Dulbecco's Modified Eagle Medium (DMEM), and fetal bovine serum were obtained from Gibco (USA). Cell Counting Kit-8, Calcein-AM/PI cell viability assay kit, BCA protein assay kit, enhanced adenosine 5′-triphosphate (ATP) assay kit, Hydrogen Peroxide Assay Kit (cat#S0038), Singlet Oxygen Sensor Green (SOSG, cat#S0067) and ROS Assay Kit (DCFH-DA, cat#S0033) were provided by Beyotime (China). HAPs (5 × 5 × 2 mm) were purchased from Sichuan Baiameng Bioactive Materials Co., Ltd (China). Bovine teeth were provided by Wenzhou Medical University Laboratory Animal Resources Center. A live/dead BacLight viability kit was obtained from Invitrogen (USA). Brain heart infusion (BHI) was purchased from OXOID (USA). MSA Medium was provided by Qingdao Hope Bio-Technogy Co., Ltd.

### 2.2 Synthesis of CP-SAP

The synthesis of CP-SAP was conducted through a series of three primary stages. First, we synthesized PDN by crosslinking G2-Lys through disulfide bonds. The specific procedure was as follows: 200 mg of G2-Lys was accurately weighed and dissolved in 10 mL of N,N-Dimethylformamide (DMF). Subsequently, 40 mg of Di (N-succinimidyl) 3,3′-Dithiodipropionate (DSP) was dissolved in 2 mL of DMF. The DSP solution was added dropwise to the G2-Lys solution, which was stirred continuously for 24 h. After stirring, the mixture was dialyzed against DMF for 4 h, followed by dialysis in deionized water (DDW) for 48 h. The PDN product was then collected by lyophilization. Secondly, 1 mg of Ce6 was dissolved in 1 mL of DMSO, and 15 mg of PDN was dissolved in 15 mL of DDW. The Ce6 solution was added dropwise to the PDN solution, and the mixture was stirred in the dark for 24 h. Following the reaction, the mixture was dialyzed in DDW for 48 h and lyophilized to obtain the dark green Ce6@PDN (CP) product. Finally, to conjugate the peptide sequence DpSpSEEKC (SAP, cysteine was for linkage) onto the CP surface, a maleimide-mercapto coupling reaction was employed. 16.913 mg (0.1 mmol) of 3-maleimidopropionic acid, 191.7 mg (1 mmol) of EDC, and 115.09 mg (1 mmol) of NHS were dissolved in 15 mL of DDW and reacted under nitrogen for 24 h to activate the carboxyl group of 3-maleimidopropionic acid. After the reaction, the precipitate was removed by centrifugation. The resulting solution was then added dropwise to 15 mL of a 1 mg/mL CP solution, which was stirred for 24 h to form Mal-CP. To this solution, 0.375 mg of SAP was added, maintaining the pH between 6.5 and 7.5. The mixture was stirred for 24 h, then dialyzed and lyophilized to yield the final CP-SAP product, which was stored for subsequent use.

### 2.3 Characterization of CP-SAP

Nuclear Magnetic Resonance Spectroscopy (^1^H NMR) (Bruker Avance III 400 MHz, GER) was used to confirm the synthesis of PDN. A UV–Vis spectrophotometer (UV–Vis, HITACHI UH5700) was used to determine the absorption spectra of free Ce6, PDN, CP, and CP-SAP. Particle size distributions and potentials of PDN, CP, and CP-SAP were tested with a Malvern particle size analyzer (Zetasizer Nano ZS-90, Malvern Panalytical). The morphological characteristics and elemental distribution of PDN, CP, and CP-SAP were examined by transmission electron microscopy (TEM) (Talos F200S, Thermo Fisher Scientific). The phosphorus content of CP-SAP in different groups was quantified by inductive coupled plasma mass spectrometer (ICP-MS) (Agilent 7850) for further evaluation of SAP grafting. The chemical groups of SAP and CP-SAP were analyzed by Fourier transform infrared spectroscopy (FTIR) spectrometer (Nicolet IS20, Thermo Scientific) in the wavelength range of 500–4000 nm.

### 2.4 Biosafety assessment

Human gingival fibroblasts (HGF) were cultured in a specialized medium designed for HGF cells, while mouse fibroblast L929 cells were maintained in complete medium consisting of DMEM, 10 % (v/v) fetal bovine serum, and 1 % (v/v) penicillin/streptomycin. All cells were cultured in a cell culture incubator at 37 °C with 5 % CO_2_.

Cytotoxicity was evaluated using the CCK-8 assay. HGF and L929 cells were seeded into 96-well plates at a density of 10,000 cells per well. After one day, these cells were co-cultured with CP and CP-SAP at concentrations ranging from 50 to 250 μg/mL for a short-term duration of one day. For long-term co-culture, cells were seeded at a density of 4000 cells per well. After 24 h, they were co-cultured with CP and CP-SAP at the same concentration range (50–250 μg/mL) for an extended duration of three days. After the co-culture period, 10 μL of CCK-8 solution was added to each well, and the incubation was carried out. The absorbance of each well at 450 nm was measured using a microplate reader (M5, Molecular Devices, USA). Cell viability was calculated using the formula: Cell viability (%) = [OD (sample) – OD (blank)]/[OD (control) – OD (blank)] × 100 %.

To assess the hemolytic effect, 1 mL of fresh blood was collected from experimental mice and centrifuged at 3000 rpm for 15 min to isolate red blood cells (RBC), which were then resuspended in 1 mL of PBS. 20 μL of RBC were mixed with 1 mL of ddH_2_O, PBS, and CP-SAP solutions with concentrations ranging from 50 to 250 μg/mL, and incubated at 37 °C for 4 h. After incubation, the solutions were centrifuged again at 3000 rpm for 15 min. The supernatant was photographed, and the optical density (OD) was measured at 542 nm. Hemolysis was calculated using the formula: Hemolysis (%) = [OD (experiment group) – OD (negative group)]/[OD (positive group) – OD (negative group)] × 100 % [36].

HGF and L929 cells (10,000 cells per well) were seeded in 96-well plates and co-cultured with CP-SAP at concentrations ranging from 50 to 250 μg/mL for 24 h. After washing with PBS, the cells were stained with Calcein AM (5 μM) and PI (5 μM) for live and dead cell staining. Cell morphology and fluorescence signals were observed using an inverted fluorescence microscope (Axio Observer3, ZEISS).

### 2.5 Adhesion capacity of CP-SAP

#### 2.5.1 The short-term adhesion capacity of CP-SAP to the HAP surface

To visualize the adhesion of CP and CP-SAP to the hydroxyapatite (HAP) surface, both were labeled with Cy5, a fluorescent dye, and adhesion properties were evaluated by fluorescence intensity under a microscope. First, 2 mL of aqueous CP and CP-SAP solutions (each at 500 μg/mL) were mixed with 50 μL of a Cy5-NHS ester solution in dimethyl sulfoxide (DMSO) at the same concentration. The mixture was stirred at room temperature for 4 h. Afterward, the solution was dialyzed with deionized water (DDW), and the product was collected after lyophilization. The surfaces of 5 × 5 × 2 mm HAP slices were sealed with nail polish, leaving circular regions of approximately 3 × 3 mm exposed at the centers. The HAP slices were then immersed in 200 μg/mL Cy5-labeled CP and CP-SAP solutions for 2, 4, 6, and 8 min. After immersion, the HAP slices were removed and rinsed with deionized water (DDW) to remove any non-adherent material. Following drying, the short-term adhesion of CP-SAP was assessed using an orthogonal fluorescence microscope (Nikon NI-B, Japan).

To quantify the amount of CP-SAP rapidly adhering to the hydroxyapatite (HAP) surface, HAP slice were immersed in 1 mL of CP-SAP aqueous solution with a concentration of 200 μg/mL. The HAP slices were also immersed for 2, 4, 6, and 8 min, respectively. Following this, the sample was centrifuged at 5000 rpm for 5 min, and the supernatant was collected. The amount of CP-SAP that failed to adhere to HAP in the short term was determined by measuring the ultraviolet absorption peak at 665 nm of the supernatant. This assessment relied on the standard curve of CP-SAP.

#### 2.5.2 The long-term retention capacity of CP-SAP to the surface of HAP

Building on the optimal short-term adhesion time (6 min) established in previous experiments, the long-term adhesion performance of CP-SAP on HAP samples was further evaluated. To simulate oral conditions and practical scenarios, HAP slices treated for 6 min were divided into three groups. One group was immersed in 1 mL of artificial saliva without laser irradiation, while the second group was placed in an acidic solution (pH 5.5) without irradiation. The third group was pretreated with a 665 nm laser (power: 214 mW/cm^2^) for 5 min, with the laser source positioned 1 cm from the HAP surface. After treatment, all samples were agitated at 37 °C and 100 rpm for 12 h, 1 day, 3 days, and 5 days. At each time point, the samples were retrieved, rinsed, and dried. Finally, fluorescence intensity on the HAP surface was observed using an orthogonal fluorescence microscope to assess the long-term retention of CP-SAP.

For more precise quantification, after achieving optimal short-term adhesion, the HAP slices were immersed in 1 mL of artificial saliva and agitated at 37 °C and 100 rpm for the same time points (12 h, 1 day, 3 days, and 5 days). Afterward, the samples were centrifuged to collect the supernatant, and the mass of CP-SAP that did not adhere to the HAP surface over the long term was determined by measuring the ultraviolet absorption peak at 665 nm of the supernatant. This calculation was based on the standard curve of CP-SAP [34].

#### 2.5.3 The in vivo adhesion capacity of CP-SAP to enamel samples

Enamel slices were extracted from bovine teeth provided by the Laboratory Animal Resources Center of Wenzhou Medical University. Using a dental drill, perforations were created on the enamel slice surfaces. The surface was then polished to leave a central area of 5 × 5 mm, with the surrounding region sealed using nail polish. To simulate early caries, the enamel slices were etched with 35 % phosphoric acid (Heraeus, GER) for 2 min, followed by rinsing, drying, and setting them aside for future use [37].

SD rats were anesthetized with isoflurane (RWD Life Science, China) and further anesthetized with an intraperitoneal injection of sodium pentobarbital to ensure deep anesthesia. The enamel slices were then sutured to the buccal side of the rats' oral cavities. After fixation, 200 μL of Cy5-labeled CP-SAP (200 μg/mL) were applied to the enamel slice surfaces, and the samples were protected from strong rinsing by saliva or other fluids for 6 min. After 24 h, the enamel slices were removed, and the adhesion of CP-SAP to the enamel surfaces in the rats' oral cavities was evaluated using an orthogonal fluorescence microscope.

### 2.6 Generation of ROS during aPDT

To quantify the hydrogen peroxide (H_2_O_2_) production by CP-SAP during aPDT, An H_2_O_2_ assay kit was used. In each well of a 96-well plate, 50 μL of CP-SAP containing 3 μg/mL of Ce6 was added, followed by the addition of 100 μL of hydrogen peroxide detection reagent. The samples were then irradiated continuously with a 665 nm laser at an output power of 43 mW/cm^2^ for 0, 1, 3, 5, and 10 min. After irradiation, the samples were left to stand in the dark for 10 min, and the optical density at 560 nm was measured using a multifunctional microplate reader.

For singlet oxygen (^1^O_2_) detection, the SOSG assay was conducted. In each well of a 96-well plate, 50 μL of CP-SAP containing 3 μg/mL of Ce6 was mixed with 100 μL of SOSG (3 μM). The samples were processed as previously described, and the fluorescence of the mixture was measured using a microplate reader with an excitation wavelength of 504 nm and an emission wavelength of 525 nm.

To measure total reactive oxygen species (ROS) generation, the DCFH-DA assay was performed. In a 96-well plate, 50 μL of CP-SAP containing 3 μg/mL of Ce6 was mixed with 100 μL of DCFH-DA (6 μM). The same procedure was followed as described earlier, and the fluorescence intensity was measured using a multifunctional microplate reader with an excitation wavelength of 488 nm and an emission wavelength of 525 nm. Samples subjected to 665 nm laser irradiation were denoted as "+ L". The control groups included PBS + L, free Ce6 + L (3 μg/mL), CP + L (3 μg/mL Ce6), and CP-SAP without laser irradiation (n = 3).

### 2.7 In vitro antimicrobial activity of CP-SAP

#### 2.7.1 CFU counting

The antimicrobial effect of CP-SAP was evaluated in vitro using the colony-forming unit (CFU) counting method, selecting three bacterial strains: *Streptococcus mutans* (*S. mutans*), *Streptococcus sanguis* (*S. sanguis*) and *Candida albicans* (*C. albicans*). Chlorhexidine (CHX) gargle was used as the positive control group. In each experiment, 950 μL of bacterial suspension of *S. mutans* and *S. sanguis* (both at a concentration of 10^9^ CFU/mL) and *C. albicans* (at a concentration of 10^8^ CFU/mL), were mixed with 50 μL of CP-SAP at a concentration of 4 mg/mL, along with other treatment groups. The resulting mixtures were transferred to Eppendorf tubes and irradiated with a 665 nm laser at an intensity of 214 mW/cm^2^ for 5 min. After irradiation, the samples were incubated in the dark for an additional 30 min. The treated mixtures were then diluted in gradients to obtain five sets of solutions with concentrations ranging from 10^6^ to 10^2^ CFU/mL. Next, 5 μL of each diluted solution was spotted onto BHI agar plates and incubated overnight at 37 °C with 5 % CO2. Finally, the resulting colonies on the plates were enumerated [36].

#### 2.7.2 Live/dead staining

First, we treated the bacterial suspension with a concentration of approximately 10^9^ CFU/mL according to the same antimicrobial procedures as the colony counting method, and then collected bacterial pellets through centrifugation. 1 mL of fluorescent dye solution containing equimolar concentrations of propidium iodide (PI) and SYTO 9 (both at 1.6 μM) was added to each sample tube. The samples were gently mixed and incubated in the dark at 37 °C for 30 min for live/dead bacterial staining. After incubation, the pellets were collected by centrifugation and resuspended in 100 μL of physiological saline. Then, 5 μL of the stained sample was placed onto a microscope slide and observed under an inverted fluorescence microscope.

#### 2.7.3 Bacterial morphology observation

First, the bacterial samples, treated using the same procedure as the colony counting method, were fixed at room temperature with 1 mL of 2.5 % glutaraldehyde. Subsequently, the samples were centrifuged and washed, then subjected to a gradient dehydration process through a series of ethanol concentrations from 30 % to 100 %, with each step lasting for 30 min. After completion of the dehydration, the samples were centrifuged again, resuspended in 100 μL of physiological saline, and dropped onto silicon wafers. After drying at room temperature, gold was sputtered onto the sample surface. Finally, we used a scanning electron microscope (SEM, model SU8010, HITACHI, Japan) to meticulously observe the samples and analyze the morphological characteristics of the bacteria in detail.

#### 2.7.4 Protein leakage and changes in ATP levels

To assess the impact of CP-SAP + L and other treatment groups on protein leakage from *S. mutans* and *S. sanguis*, we employed a BCA protein assay kit. Initially, we processed the bacteria using the same antimicrobial procedures as the colony counting method and collected the supernatant through centrifugation. Subsequently, we quantified the protein concentration in the supernatant using the BCA assay kit and measured the absorbance at a wavelength of 560 nm with a multifunctional microplate reader. To assess changes in intracellular ATP levels, we processed the bacteria using the same antimicrobial procedures as the colony counting method, collected bacterial pellets through centrifugation, and then lysed the bacteria using 200 μL of lysis buffer, followed by a second centrifugation at 4 °C and 12,000 g for 5 min to collect the supernatant. Finally, in a 96-well plate, we added 100 μL of ATP detection working solution, followed by 20 μL of the supernatant to be tested, and measured the chemiluminescence of the mixture using the luminometer function of the microplate reader.

#### 2.7.5 In vitro inhibition of biofilm formation

In a 24-well plate, we placed round coverslip (14 mm) and added 450 μL of bacterial suspension with a concentration of 10^9^ CFU/mL, as well as 50 μL of CP-SAP at a concentration of 2 mg/mL and other treatment group solutions. Then, it was irradiated with a laser of 665 nm wavelength and 214 mW/cm^2^ power density for 5 min. After 48 h, the biofilm had formed. We gently washed the biofilm twice with PBS, then added 500 μL of a mixed fluorescent dye of PI and SYTO-9 for staining for 30 min. After staining, the dye was aspirated and the biofilm was washed again. Finally, the fluorescence of the biofilm was observed using Confocal Laser Scanning Microscopy (CLSM, LSM900, ZEISS, GER).

Additionally, to quantitatively assess the inhibitory effect on biofilm formation, we employed the crystal violet staining method. The specific steps were as follows: In a 96-well plate, 50 μL of bacterial suspension with a concentration of 10^9^ CFU/mL was mixed with 50 μL of CP-SAP solution prepared with BHI medium at a concentration of 400 μg/mL, along with other treatment group solutions. The mixture was then subjected to the same laser irradiation parameters and duration as mentioned earlier. After irradiation, the mixture was co-incubated for 48 h. Subsequently, the samples were fixed with methanol, stained with 0.1 % crystal violet for 30 min, then washed and photographed to document the remaining biofilm. After photography, the residual biofilm was dissolved using a 33 % acetic acid solution, and the absorbance at 596 nm was measured with a microplate reader. The survival rate of the biofilm was calculated using the formula: Biofilm survival rate = OD_596_ (experiment group)/OD_596_ (PBS group) × 100 % [38].

### 2.8 In vitro remineralization assay of CP-SAP

In order to prepare enamel samples, we obtained healthy bovine incisors from the Laboratory Animal Resources Center of Wenzhou Medical University. We utilized a hard tissue cutter (Struers Minitom, Denmark) to meticulously cut and polish enamel slices to dimensions of approximately 1 × 1 cm. These samples were then subjected to a 2-min etching process with 35 % phosphoric acid. After etching, the samples were rinsed with DDW for 1 min and air-dried to obtain demineralized enamel samples [37].

Next, we uniformly applied 500 μL of PDN, CP, CP-SAP aqueous solutions (200 μg/mL), as well as Fluoride varnish, to the etched enamel samples and let them act for 5 min. The samples were then soaked in 1 mL of artificial saliva and incubated in a shaker at 37 °C, 100 rpm, for five days. During the artificial saliva incubation period, the samples were treated once a day with the aforementioned materials. After five days, the samples were sonicated, then rinsed with DDW and air-dried at room temperature. The surfaces and cross-sections of the etched, untreated samples and the etched, treated samples were analyzed using scanning electron microscopy (SEM). The calcium-to-phosphorus ratios in the original enamel, CP-SAP-treated enamel, Fluoride varnish-treated enamel, and etched enamel were determined using energy-dispersive X-ray spectroscopy (EDX). The crystal structure of the newly formed minerals was characterized by X-ray diffraction (XRD) analysis. The impact of the treatments on the surface hardness of the enamel was evaluated using a calibrated Vickers hardness tester. Finally, the thickness variations of the remineralized layer on the enamel surfaces post-CP-SAP application were examined using SEM after treatment periods of 10, 12, and 14 days.

### 2.9 Animal experiment

First, 20 SD 6-week-old male rats were separated into four groups: PBS, CHX, free Ce6 + L, and CP-SAP + L. To avoid the influence of endogenous oral bacteria, all SD rats were constantly administered antibiotic water with penicillin (200 μg/mL), streptomycin (1500 μg/mL), and antibiotic-added food on days 1–3 [19]. After three days, plaque was sampled from the dental surface using a sterile cotton swab, followed by ultrasonic treatment and gradient dilution. The samples were then plated on MSA selective medium and incubated for 24 h in an anaerobic environment at 37 °C to assess the effectiveness of antibiotic feeding. From days 4–6, 200 μL of *S. mutans* bacterial suspension (1 × 10^8^ CFU/mL) was inoculated onto each rat's teeth (daily at 8:00 a.m., daily at 12:00 noon, and daily at 18:00 p.m.). The effectiveness of the bacterial inoculation was then verified using the method described above. The rats were fasted for 30 min before taking the bacteria and after inoculation with the bacteria. The rats were provided with cariogenic chow (DYET# 160323, Dyets Biotechnology (Wu Xi) Co., Ltd) and 5 % sucrose water starting from the time they were infected with the bacteria until the completion of the experiment, to cause the development of caries. After confirming the effectiveness of antibiotic feeding and bacterial inoculation, the treatment process was initiated in the rats. Following isoflurane-induced anesthesia, the bilateral maxillary molar regions were treated with 200 μL of PBS, CHX, free Ce6, and CP-SAP. The 665 nm laser (power: 214 mW/cm^2^) was applied to the maxillary teeth of the rats, with a custom-designed transparent polycarbonate (PC) plate used to fix the laser source at a consistent distance of 1 cm from the surface of the second maxillary molar. Administered daily from day 7 through day 21, the therapy concluded on day 21. All rats fasted for 30 min before and after each treatment. Body weight data were recorded at intervals on days 1, 4, 7, 9, 15, and 21. Furthermore, on days 4, 6, 9, 15, and 21 following the treatment, a sterile cotton swab was used to collect plaque from the dental surface for 30 s after the procedure. The collected microorganisms were then placed into 1 mL of saline solution, sonicated, serially diluted, spread onto MSA selective medium, and incubated for 24 h. Subsequently, bacterial growth was monitored, and the quantity of bacteria was recorded. After the 21-day treatment period, the rats were humanely sacrificed via the inhalation of an excessive amount of isoflurane. The maxilla, teeth, oral mucosa, tongue, blood, and major organs were harvested. A micro-CT (Brucker Skyscan1276, GER) was used to examine the degree of dental caries. The teeth were stained with murexide, and after staining, they were photographed and recorded using a stereomicroscope (SMZ800N Nikon, Japan) and the Keyes scoring was used to score them. The caries scores were jointly determined by one inspector responsible for calibration and two additional inspectors.

### 2.10 Histopathological analysis

H&E staining was conducted on the gingival mucosa, tongue, and major organs within the rat's oral cavity. Additionally, blood biochemical analyses were performed to assess the biocompatibility of CP-SAP for its application within the rat's oral cavity.

### 2.11 Statistical analysis

Each experiment was performed at least three times. Results were presented as mean ± standard deviation (SD) and were analyzed using ANOVA. Statistical significance was assessed using an unpaired Student's t-test. Data were considered to be statistically significant at the following p-values: ∗p < 0.05, ∗∗p < 0.01, ∗∗∗p < 0.001, ∗∗∗∗p < 0.0001.

## 3 Results and discussion

### 3.1 CP-SAP synthesis and characterization

**Fig. 1:**
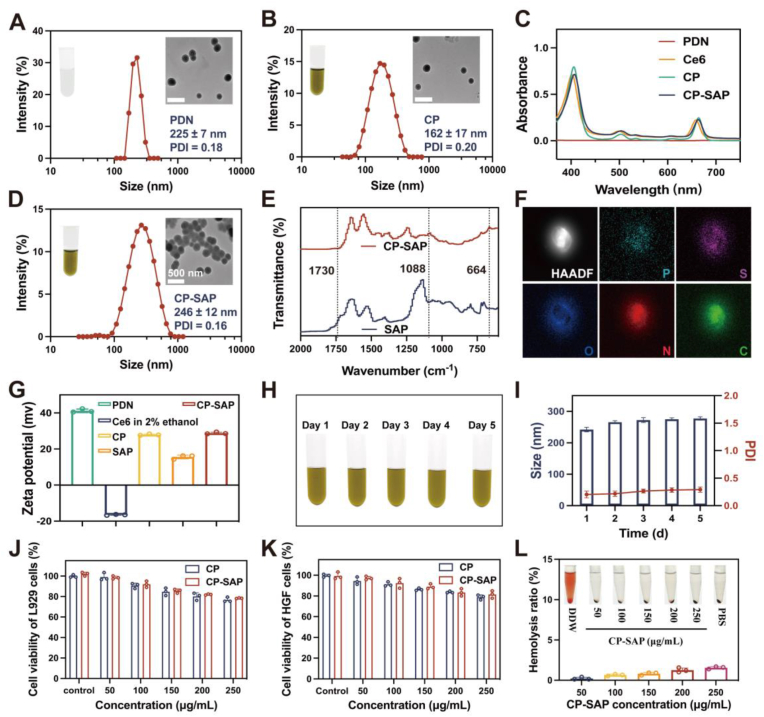
(A–B, D) Particle size and TEM images of PDN, CP, and CP-SAP. (C) UV–vis absorption spectra for CP and various materials. (E) FTIR spectra of SAP and CP-SAP. (F) Elemental mapping of CP-SAP. (G) Zeta potential of various materials. (H, I) Stability of CP-SAP over 5 days at 37 °C. (J, K) Using the CCK-8 assay to evaluate the cytotoxicity of L929 and HGF cells after treatment with CP-SAP (50–250 μg/mL) for 24 h. (L) Hemolytic behavior of CP-SAP (50–250 μg/mL). Data are presented as mean ± standard deviation (SD) (n = 3).

Successful construction and characterization of the CP-SAP was achieved via a complex multi-stage process. G2-Lys, a second-generation polylysine dendrimer with a POSS core, was cross-linked with the DSP to form a colorless, transparent PDN solution with a size of 225 ± 7 nm and a PDI of 0.18 (Fig. 1A). The ^1^H NMR spectra revealed new chemical shifts attributed to the methylene group of the DSP crosslinker near 2.51–2.71 ppm [39] (Fig. S1), confirming the successful incorporation of DSP into the peptide dendrimer structure and the successful synthesis of PDN. In order to encapsulate Ce6 into PDN, three different Ce6-to-PDN feeding ratios (5 %, 6.67 %, and 10 % w/w) were systematically tested to determine the optimal ratio. The drug loading capacity and encapsulation efficiency of Ce6 increased as the feeding ratio rose. However, the optimal size and stability of the Ce6-loaded PDN (Ce6@PDN, referred to as CP) were observed at a Ce6 feeding ratio of 6.67 %, with a drug loading capacity of 6.35 % and an encapsulation efficiency of 89.42 % (Table S2). This ratio was selected for the preparation of CP. After encapsulation, CP exhibited a characteristic spherical morphology with a PDI of 0.20 and an average diameter of 162 ± 17 nm (Fig. 1B), which was smaller than that of the PDN. This size reduction was attributed to the encapsulation of Ce6, which made the hydrophobic core of PDN denser and more stable. The aqueous solution of CP exhibited a uniform deep green color without any precipitation (Fig. S3), contrasting with free Ce6, which aggregated and sank to the bottom of the tube. This result indicated that using PDN as a carrier significantly enhanced the solubility of Ce6 in the aqueous phase, laying the foundation for the practical application of Ce6 in physiological environments. Furthermore, the UV–visible absorption spectrum of CP (Fig. 1C) showed a characteristic absorption peak of Ce6 at 665 nm, confirming that Ce6 was effectively encapsulated within PDN and that CP was successfully synthesized. To improve the adhesion properties of CP, a further grafting of SAP was performed. Four different SAP-to-CP feeding ratios (2 %, 2.5 %, 3.33 %, and 5 % w/w) were evaluated. Increasing the feeding ratio to 3.33 % resulted in destabilization of the solution with a PDI >0.30, and the stability decreased further with higher SAP feeding ratios. The P content in CP-SAP was quantified using inductively coupled plasma mass spectrometry (ICP-MS), and the grafting rate was determined based on the molecular weight of SAP. The grafting rate increased with the feeding ratio, but approached saturation at a ratio of 2.5 % (Table S4). Therefore, the 2.5 % feeding ratio was selected to prepare the final CP-SAP nanosystem. The resulting CP-SAP contained 47.72 μg of SAP per 1 mg of CP-SAP, with a grafting rate of 91.6 %. The final CP-SAP had an average diameter of 246 ± 12 nm and a PDI of 0.16 (Fig. 1D). Fourier transform infrared (FTIR) analysis confirmed successful grafting of SAP. A small peak at 1730 cm^−1^, corresponding to the sulfhydryl group of cysteine, was observed in the SAP spectrum, but it disappeared in the CP-SAP spectrum. Two new peaks at 1088 and 664 cm^−1^ were observed in the CP-SAP spectrum, attributed to thioether bonding during the grafting process (Fig. 1E) [40]. The elemental mapping diagram (Fig. 1F) showed a homogeneous distribution of phosphorus (P) atoms across the CP-SAP, further confirming successful grafting. The potential of the PDN was reduced from 40.8 mV to 27.82 mV after the encapsulation of negatively charged Ce6; however, it increased slightly to 28.59 mV following the successful grafting of positively charged SAP (Fig. 1G). Additionally, CP-SAP demonstrated excellent stability, as its color, particle size, and PDI remained stable after 5 days of incubation at room temperature (Fig. 1H and I).

Biosafety is a crucial concern for in vivo applications. Results showed that cell viability for both L929 and HGF cells remained above 80 % even after 1 or 3 days of incubation with 200 μg/mL of CP-SAP (Fig. 1J, K, S5). Moreover, CP-SAP exhibited a hemolysis rate of less than 5 % across concentrations ranging from 50 to 250 μg/mL (Fig. 1L). Based on these results, 200 μg/mL was chosen as the working concentration for subsequent experiments due to its favorable biocompatibility and hemocompatibility. Furthermore, the light green color of CP-SAP (at 200 μg/mL) was lighter than CHX, which could potentially improve patient acceptability and clinical applicability (Fig. S6).

In conclusion, CP-SAP has been successfully synthesized and characterized, exhibiting excellent stability and favorable biosafety profiles. These properties make CP-SAP a promising candidate for use as a gargle solution, with potential for clinical applications.

### 3.2 Wet adhesion capacity of CP-SAP

Dental enamel is primarily composed of hydroxyapatite (HAP) crystals (∼96 wt%) [41], which share similar chemical composition and crystal structure with enamel. Due to these similarities, HAP powder and HAP slices have been widely used as models for enamel in oral-related research [42,43]. Therefore, in this study, HAP slices were selected for adhesion experiments.

**Fig. 2:**
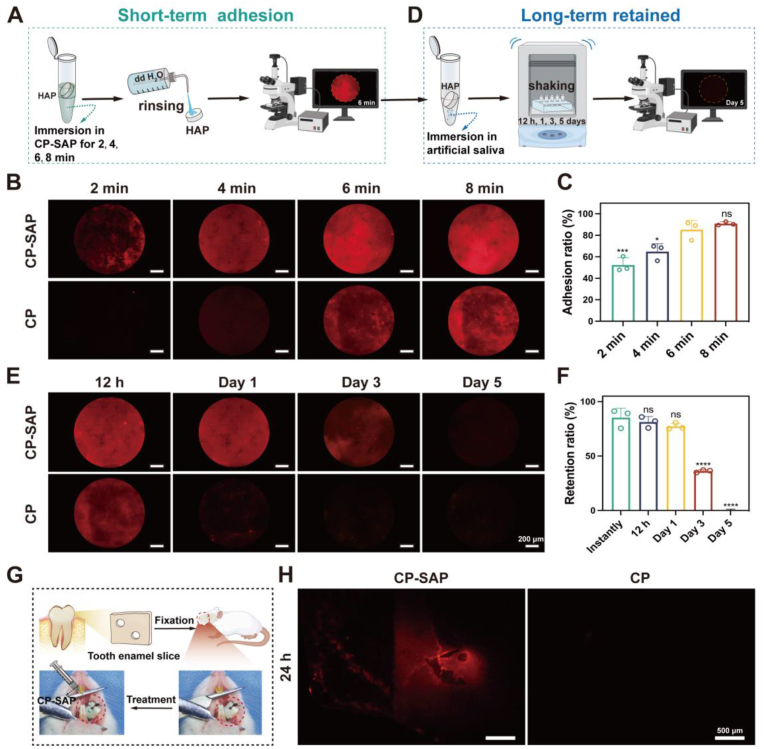
(A) Schematic of in-vitro short-term adhesion capability of CP-SAP. (B, C) Short-term adhesion capacity of CP-SAP. (D) Schematic of in-vitro long-term retention capacity of CP-SAP. (E, F) CP-SAP retention capacity in artificial saliva. (G) Schematic of CP-SAP adhesion capability in vivo in rats. (H) Adhesion capacity of CP-SAP in vivo. Data are presented as mean ± SD, (n = 3), ∗p < 0.05, ∗∗∗p < 0.001, ∗∗∗∗p < 0.0001, ns: no significance. Comparisons are between the 6-min group and other groups and Instantly group and other groups.

CP and CP-SAP were labeled with the fluorescent dye Cy5 for subsequent. First, orthogonal fluorescence microscopy was used to observe the rapid adhesion of CP-SAP to the HAP surface (Fig. 2A). The results showed that CP-SAP demonstrated faster and stronger adhesion compared to CP, as evidenced by the more intense red fluorescence distribution on the HAP surface in the CP-SAP-treated group after the same treatment duration (Fig. 2B). Quantitative analysis of the supernatant using UV–visible spectroscopy further confirmed that CP-SAP achieved persistent and adequate adhesion to the HAP surface after 6 min of treatment (Fig. 2C). Based on these findings, a 6-min adhesion time was chosen for subsequent testing of the long-term retention potential of CP-SAP.

The long-term adhesion of CP-SAP to HAP was further investigated using orthogonal fluorescence microscopy (Fig. 2D). The results revealed that CP-SAP maintained vivid red fluorescence even after 24 h of exposure to artificial saliva, indicating strong long-term adhesion. In contrast, the CP group showed significantly dimmer fluorescence (Fig. 2E). Quantitative measurements also demonstrated that CP-SAP displayed 75.89 % retention at 24 h (Fig. 2F), further confirming its excellent long-term adhesion capacity. Furthermore, a range of environmental changes, including a decrease in pH to 5.5 and exposure to 5 min of laser irradiation, did not significantly affect the long-term adhesion capability of CP-SAP to HAP surface (Figs. S7 and 8).

Considering that CP-SAP will be subject to continuous saliva flushing and various functional movements within the oral cavity during practical application, which could significantly affect its adhesion performance, we performed in vivo animal experiments to further validate the adhesion capacity of the CP-SAP system in the oral environment (Fig. 2G). Fluorescence microscopy results showed that significant red fluorescence remained on the surface of bovine enamel slices fixed in the oral cavity of rat models after 24 h, which was notably higher than the retention observed in the CP group (Fig. 2H).

In conclusion, the superior adhesion capabilities of CP-SAP are primarily attributed to the wet adhesion properties of SAP. These properties arise from the unique amino acid sequences and charge characteristics within SAP's molecular structure, particularly the electrostatic interactions between the phosphoryl side chains of phosphorylated serine (pS) and the Ca^2^⁺ ions present on the surfaces of HAP or teeth [33]. Our studies demonstrate that CP-SAP can achieve stable adhesion to the HAP surface in as little as 6 min, maintaining adhesion for more than 24 h under both in vitro and in vivo conditions. We believe that the persistent adhesion of CP-SAP in the moist environment of the oral cavity forms the foundation for its potential applications in antibacterial treatment and remineralization therapy.

### 3.3 ROS generation capacity of CP-SAP

**Fig. 3:**
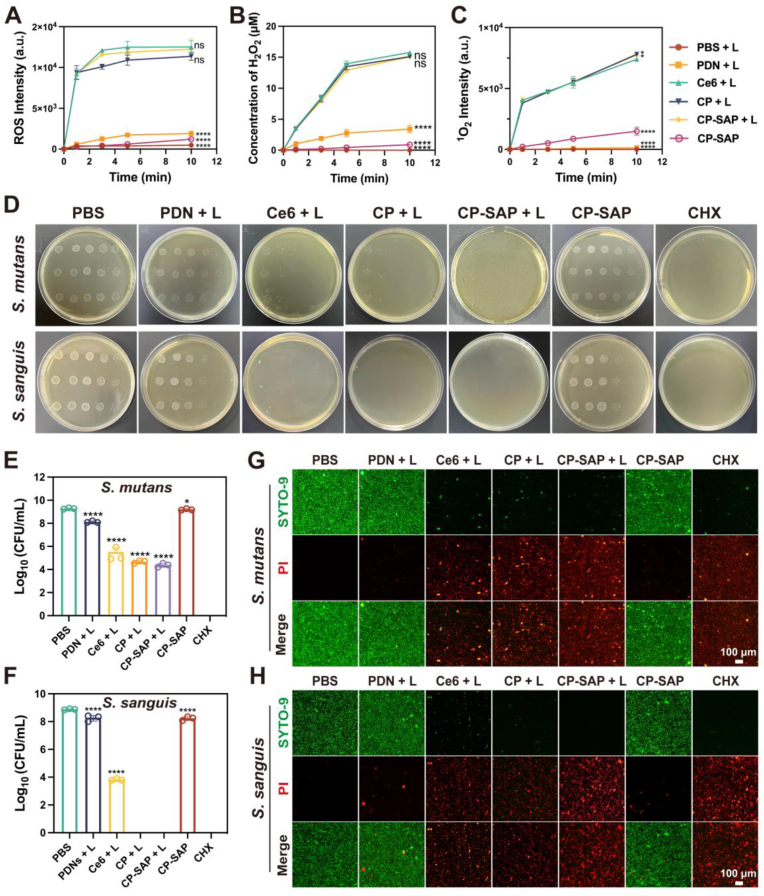
(A–C) Generation profiles of ROS, H_2_O_2_, and ^1^O_2_ for each group (L: 5 min of laser irradiation). (D) Representative plate images of *S. mutans* and *S. sanguis* after various treatments with corresponding surviving CFUs of (E) *S. mutans* and (F) *S. sanguis*. (G, H) Representative live/dead staining images of planktonic *S. mutans* and S. sanguis after various treatments. Data are presented as mean ± SD, (n = 3), ∗p < 0.05, ∗∗∗p < 0.001, ∗∗∗∗p < 0.0001, ns: no significance. Comparisons are between the Ce6 + L group and other groups, PBS groups and other groups.

As previously mentioned, aPDT is a novel antimicrobial technology that uses laser to activate photosensitizers and consequently generate ROS to kill bacteria. In our examination of the generation of various types of ROS, we observed that the laser rapidly activated CP-SAP, whereas the functionalization modification did not reduce the photosensitivity of Ce6. After 10 min of irradiation with a 665 nm laser, there was no significant difference in the total ROS production among the CP + L, CP-SAP + L, and free Ce6 + L groups (Fig. 3A), and the increase in total ROS levels tended to plateau after 5 min of irradiation. The levels of H_2_O_2_ production observed for CP + L and CP-SAP + L also exhibited trends of increase that were very similar to that observed for the free Ce6 group (Fig. 3B). Singlet oxygen, a type of ROS that responds to aPDT, is known as a potent oxidant that is capable of oxidizing a variety of molecules comprising bacteria to further drive bacterial cell death [44]. As demonstrated in Fig. 3C, CP + L and CP-SAP + L exhibited a slightly larger capacity for singlet oxygen production than that of free Ce6 + L. This was attributable to the more homogeneous and stable distribution and smaller size of CP and CP-SAP compared to that of the free Ce6 group, which resulted in a larger surface area-to-volume ratio and ultimately enhanced the formation of singlet oxygen [45,46].

In general, as the duration of laser exposure increases, the level of ROS generated by CP-SAP + L rises. Consequently, extending the laser exposure time may enhance its antimicrobial efficacy. However, based on the rate of ROS generation and considering the practicality of clinical procedures, patient comfort, and the need to minimize damage to normal tissues, we selected a 5-min irradiation duration for subsequent experiments to achieve a balance between efficacy and safety.

### 3.4 In-vitro antimicrobial activity of CP-SAP

We have demonstrated that CP-SAP can generate various types of reactive oxygen species (ROS), and we hypothesized that photodynamic therapy mediated by CP-SAP could effectively kill cariogenic bacteria through ROS production. To test this hypothesis, *S. mutans* and *S. sanguis* were selected as bacterial models to evaluate their antibacterial effects. *S. mutans* is a key pathogen in dental caries [47], while *S. sanguis* is an important early colonizer of the dental surface [48]. As demonstrated in Fig. 3D–S9**,** CP-SAP + L exhibited significant antibacterial activity against both *S. mutans* and *S. sanguis.* The antibacterial effect of CP-SAP + L on *S. sanguis* was comparable to that of the gold standard of the antimicrobial gargle—chlorhexidine (CHX), with nearly 8.8 orders of magnitude of *S. sanguis* eliminated (Fig. 3E and F). Moreover, the antibacterial effect of the CP-SAP + L group against both cariogenic bacteria was higher than those of the free Ce6 + L and CP + L groups. This enhanced effect is attributed to the positive zeta potentials of both PDN and SAP, with the zeta potential of CP-SAP being greater than that of CP and Free Ce6 (CP-SAP > CP > Free Ce6). This promotes electrostatic interactions with negatively charged bacterial membranes, ultimately leading to bacterial cell death [49]. Additionally, the self-assembly behaviors of CP-SAP enhance the photostability of the encapsulated Ce6. The results of the live/dead bacterial staining experiment shown in Fig. 3G, H further confirmed the significant antibacterial effects of the CP-SAP + L group on *S. mutans* and *S. sanguis*. We observed that both types of bacteria in the CP-SAP + L group exhibited strong red fluorescence and weak green fluorescence. This phenomenon indicates significant membrane damage, which allows the propidium iodide (PI) dye to enter the bacteria and emit red fluorescence upon binding to DNA. These findings highlight the potential of CP-SAP + L in antibacterial therapy.

**Fig. 4:**
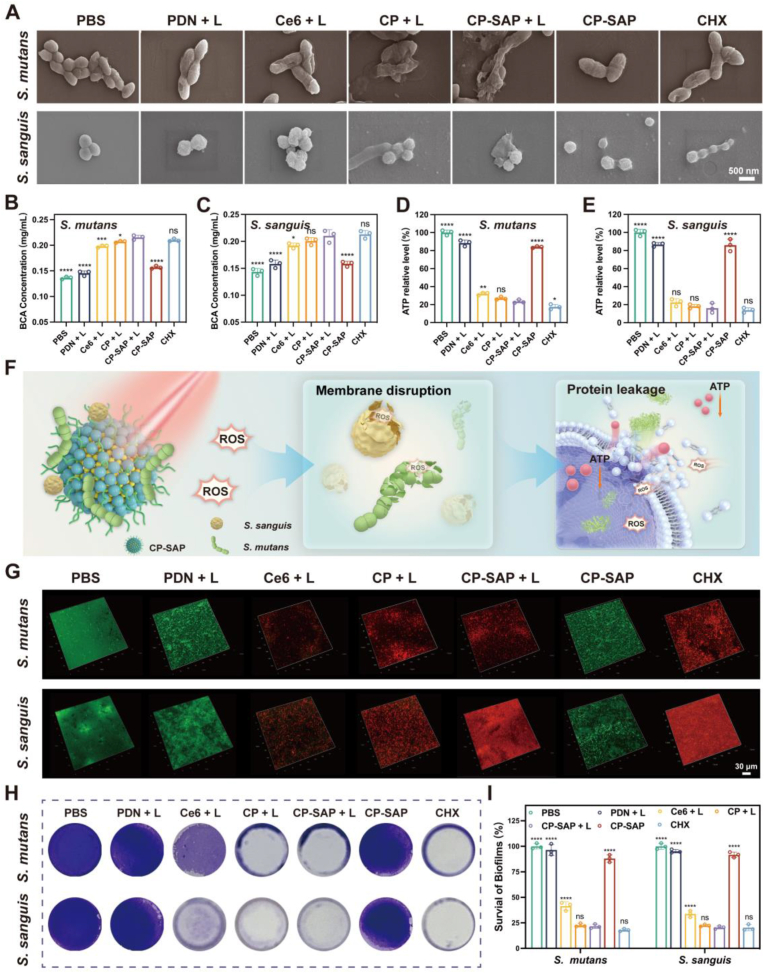
(A) SEM images of *S. mutans* and *S. sanguis* after various treatments. (B, C) Protein leakage in *S. mutans* and *S. sanguis* after various treatments. (D, E) ATP level changes of *S. mutans* and *S. sanguis* after various treatments. (F) Schematic of the antimicrobial mechanism of CP-SAP + L. (G) Live/Dead staining of biofilm formation inhibition. (H) Crystal violet staining of *S. mutans* and *S. sanguis* biofilms in different treatment groups. (I) Residual bioflim rates of *S. mutans* and *S. sanguis* in different treatment groups. Data are presented as mean ± SD, (n = 3), ∗p < 0.05, ∗∗∗p < 0.001, ∗∗∗∗p < 0.0001, ns: no significance. Comparisons are between the CP-SAP + L group and other groups.

To delve into the mechanisms underlying the antibacterial effects, we initially analyzed the surface morphology of two types of bacteria under different treatment conditions using SEM, as shown in Fig. 4A, We observed that bacteria treated with PBS maintained a smooth surface and intact cellular structure, while those treated with CP-SAP + L exhibited significant shrinkage and morphological collapse, indicating the destruction of bacterial cell membranes. As shown in Fig. 4B and C, where CP-SAP + L treatment significantly increased the protein leakage from bacteria. This further confirmed the occurrence of cell membrane damage and cell death. Furthermore, changes in ATP levels provided another important perspective for assessing the physiological state of bacteria. As a core indicator of cellular energy metabolism, a decrease in ATP levels indicated that cellular energy metabolism has been compromised [50]. We found that the ATP levels of both types of bacteria treated with CP-SAP + L significantly decreased, with the ATP levels of *S. mutans* and *S. sanguis* dropping to 23.4 ± 1.97 % and 16.11 ± 5.35 % of the levels in the PBS-treated group, respectively (Fig. 4D and E). This result indicated that CP-SAP + L severely affected the metabolism and vitality of bacteria. In summary, aPDT mediated by CP-SAP can generate ROS, which destroy bacterial membranes, trigger protein leakage, and disrupt the synthesis and maintenance of ATP, ultimately leading to bacterial cell death (Fig. 4F).

Dental plaque plays a decisive role in the initiation and progression of dental caries [51], and its biofilm structure endows bacteria with stronger resistance to antimicrobial agents compared to planktonic bacteria [52,53]. In light of this, we investigated the effects of CP-SAP + L on the formation of biofilms by *S. mutans* and *S. sanguis* using CLSM to assess its inhibitory effects. In Fig. 4G, the biofilms in the PBS group maintained their integrity, with uniformly distributed green fluorescence signals indicating stable biofilm structures. In contrast, the free Ce6 + L group only exhibited weak red fluorescence signals, likely due to the poor water solubility of free Ce6, which results in insufficient stability in aqueous solutions, and its negative charge characteristic, which hinders its ability to penetrate the negatively charged biofilms, thus leading to a relatively weak inhibitory effect on biofilm formation [36]. Compared with the Ce6 + L group, the CP + L group and the CP-SAP + L group showed significant red fluorescence signals, indicating their obvious inhibitory effects on biofilm formation. This significant outcome is attributed to the effective encapsulation of Ce6 by PDN, which significantly enhances its water solubility and transforms the material's negative charge to positive. This change in charge enhances the penetrating ability of Ce6 into the biofilm, thereby improving its efficacy in inhibiting biofilm formation. Additionally, we employed the crystal violet staining method to quantitatively assess the biofilm formation capabilities of each group. As shown in Fig. 4H and I, compared to the Ce6 + L group, the CP-SAP + L group significantly inhibited the formation of immature biofilms of *S. mutans* and *S. sanguis*, with biofilm residual rates being only 21.57 ± 2.08 % and 20.42 ± 1.4 % of the PBS group, respectively. This result was consistent with the findings we previously discussed. Notably, there was no statistically significant difference in the inhibitory effect of the CP-SAP + L group compared to the positive control group treated with CHX, confirming the potent ability of CP-SAP + L to inhibit biofilm formation.

Recent studies have shown that *C. albicans* can interact with *S. mutans* to form mixed-species biofilms, thereby exacerbating the progression of dental caries [54]. In light of this, we further evaluated the antimicrobial effects of CP-SAP + L on *C. albicans*. The results demonstrated that CP-SAP + L effectively inhibited both the growth and biofilm formation of *C. albicans* (Figs. S9, 10, 11), highlighting its broad-spectrum antimicrobial potential. In conclusion, these findings suggest that CP-SAP + L is a promising antimicrobial strategy for caries prevention.

### 3.5 In-vitro remineralization ability of CP-SAP

**Fig. 5:**
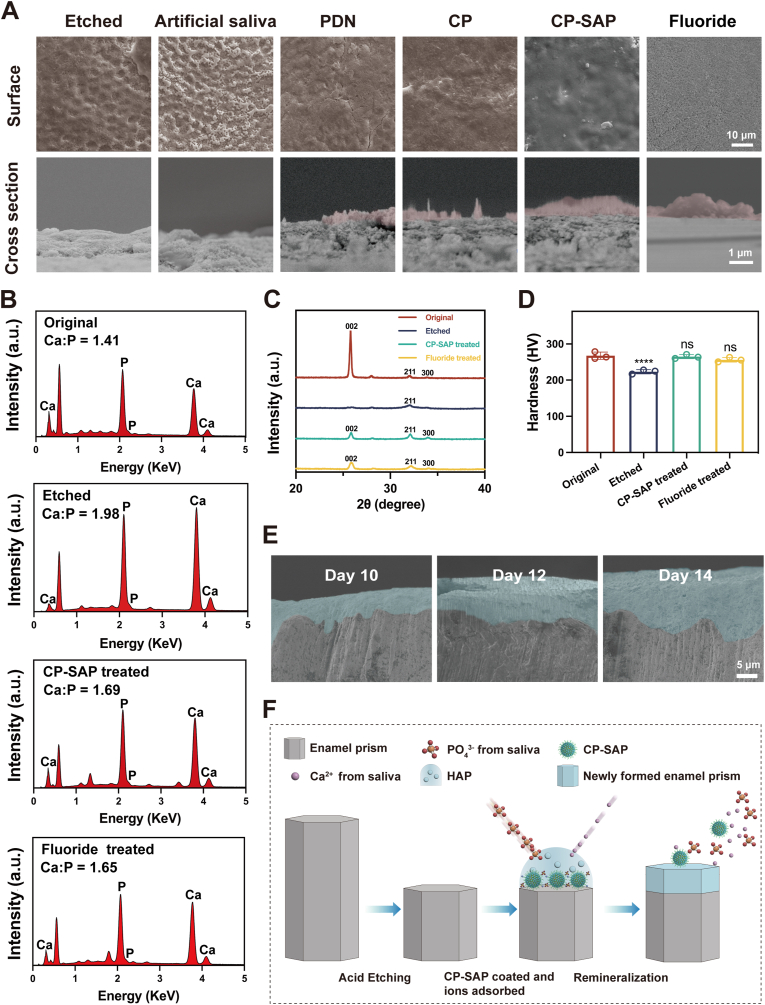
(A) SEM images of the surface and cross-section of etched enamel and enamel treated with various materials for 5 days; the pink area indicates the newly formed layer. (B) EDX spectra, (C) XRD patterns, and (D) Vickers hardness values of original, etched, and CP-SAP and fluoride-treated enamel after 5 days. (E) SEM images of enamel cross-section treated with CP-SAP for 10, 12, and 14 days. (F) Schematic of the mechanism by which CP-SAP promotes enamel remineralization. Data are presented as mean ± standard deviation (n = 3), ∗∗∗∗p < 0.0001, ns: no significance. Comparisons are made between the original enamel group and other groups.

To evaluate the remineralization effect of CP-SAP on demineralized dental hard tissues, we conducted in vitro experiments, with Fluoride varnish used as the positive control. As shown in Fig. 5A, the enamel surface after acid etching exhibited a characteristic “honeycomb” structure under the microscope. This structure arises from the higher inorganic content in enamel rods compared to interrod enamel, making the rods more susceptible to acid etching. This etching process creates a height difference between the rods and interrod enamel, resulting in the honeycomb-like appearance. This confirmed the successful preparation of the acid-etched samples. Consistent with previous research [55], pure artificial saliva demonstrated a certain degree of remineralization capability. Although the surface remained rough, small deposits began to cover the honeycomb-like structure compared to the acid-etched group. After treatment with PDN and CP, the etched enamel surface became smoother, and a relatively sparse crystalline remineralization layer was observed on the cross-section, indicating the formation of a thin new remineralized layer. This is because PDN acts as a nucleation template that attracts phosphate ions from saliva, promoting remineralization. However, due to the limited retention of PDN and CP on the enamel surface, their remineralization effects were somewhat restricted. Notably, after treatment with CP-SAP, an orderly arranged needle-like remineralized layer was observed in the enamel cross-section, with no gaps between the new layer and the original enamel. This can be attributed to the superior adhesive properties of SAP, which enable the material to adhere firmly to the etched enamel surface, even under the conditions of salivary rinse, providing long-term remineralization capabilities. Additionally, SAP itself promotes remineralization. In the positive control group, a clear crystalline structure was observed on the enamel surface, but only disordered deposits were seen in the cross-section.

To further investigate the structure of the remineralized layer induced by CP-SAP, we performed energy-dispersive X-ray spectroscopy (EDX) to determine the Ca/P ratios of the original enamel, etched enamel, and enamel treated with CP-SAP and Fluoride varnish. The Ca/P ratios for these four samples were 1.41, 1.98, 1.69, and 1.65, respectively (Fig. 5B). After treatment with CP-SAP and Fluoride, the newly formed remineralized layer on the enamel surface had a Ca/P ratio similar to that of HAP in natural tooth enamel (Ca/P ratio = 1.67) [56]. This indicates that both CP-SAP and Fluoride treatments can produce a crystal structure similar to HAP after remineralization. Furthermore, X-ray diffraction (XRD) analysis revealed no diffraction peaks in the etched group at 2θ = 25.8° (002) (Fig. 5C). However, in the groups treated with CP-SAP and Fluoride, diffraction peaks were observed at 2θ = 25.8° (002), 31.8° (211), and 32.8° (300), which were similar to those of the original enamel. These findings suggest that new crystals were formed with a high degree of enamel crystal orientation [57]. Additionally, after 5 days of treatment with CP-SAP and fluoride, the hardness of the enamel surface returned to its original level (Fig. 5D). To further explore the prolonged remineralization effects, we extended the treatment duration to 10, 12, and 14 days. SEM results (Fig. 5E) revealed that the thickness of the nascent mineral layer on the enamel surface increased with the duration of treatment, indicating continuous remineralization.

In summary, CP-SAP significantly facilitated the in situ remineralization of demineralized dental hard tissues and successfully restored their original mechanical properties, with remineralization effects comparable to those of commercially available Fluoride varnish. This achievement is attributed to the abundant functional groups on the surfaces of PDN and SAP, including amino, carboxyl, and phosphate groups, which not only provide binding sites for calcium and phosphate ions in saliva but also act as nucleation templates to stabilize calcium and phosphate ions. These ions then further react to form well-organized HAP crystals in an orderly manner (Fig. 5F), thereby effectively promoting the remineralization process.

### 3.6 In-vivo dental caries prevention effectiveness of CP-SAP

**Fig. 6:**
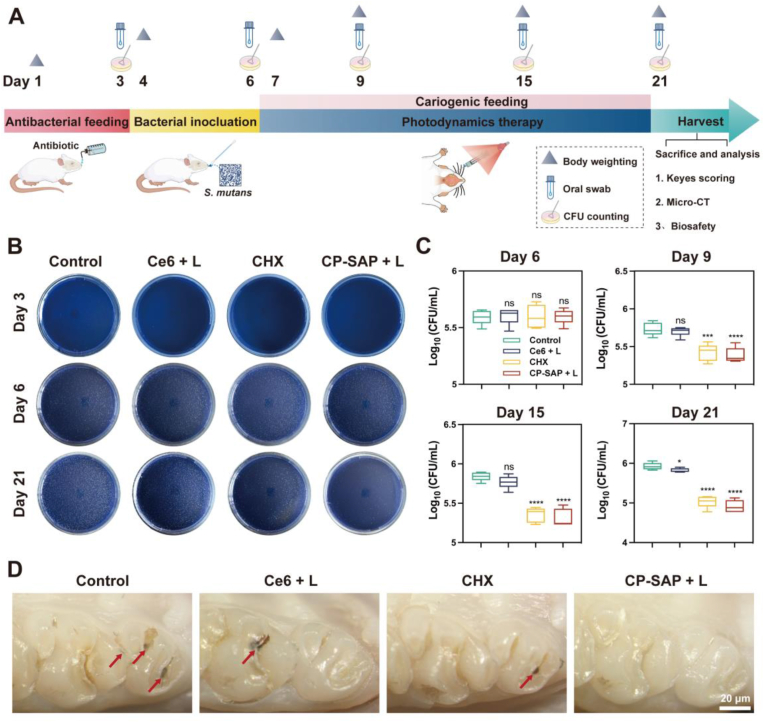
(A) Experimental design and treatment protocols for animal models. (B) Representative images of surviving bacterial colonies on MSA plates from different treatment groups (PBS, Ce6 + L, CHX, CP-SAP + L) at days 3, 6, and 21. (C) CFU counts of surviving bacterial colonies in each group on days 6, 9, 15, and 21. (D) Representative photographs of rat molars from each group, examined under a stereo-microscope at the end of the 21-day treatment period. Data are presented as mean ± SD, (n = 3), ∗∗∗p < 0.001, ∗∗∗∗p < 0.0001, ns: no significance. Comparisons are between the control and other groups.

To confirm the effectiveness of CP-SAP + L in caries prevention, we produced and established a rat caries model using the procedure detailed in Fig. 6A. Starting from Day 7, CP-SAP + L and other treatments were administered daily, with CHX (the gold standard oral antimicrobial agent) serving as the positive control. During the in-vivo experiments in rats, oral sampling and CFU assays were performed on a regular basis to monitor the population levels of caries-causing microorganisms. The oral sampling results on Day 3 revealed no bacterial colonies on selective media, indicating that endogenous oral microorganisms were effectively suppressed after antibiotic treatment. After 3 d of continuous inoculation with *S. mutans*, i.e., 6 d after the establishment of the model, the levels of colonization in each group were nearly equal, with no significant difference between the groups (p > 0.05, Fig. 6B); this suggests that caries-causing bacterial infections in the mouths of the rats were sufficiently similarly established in all groups. Throughout the treatment period, the number of bacteria in the CP-SAP + L and CHX treatment groups consistently decreased with prolonged treatment time. After 2 weeks of CP-SAP + L treatment, over 90 % of the cariogenic bacteria were eliminated (reduction of 1 log unit, Fig. 6C) and the antimicrobial rate was higher than that for the CHX gargle. Alternatively, the limited adhesion and water solubility of the free Ce6 + L group effectively restricted its efficacy, achieving only a 12.3 % antibacterial rate. These results indicate that CP-SAP + L has a high level of antimicrobial activity in vivo. Consequently, CP-SAP + L would be highly effective in preventing the onset and progression of caries. Maxillary teeth were selected from each group for further analysis. Dark brown caries were observed on the occlusal surfaces of the PBS control, free Ce6 + L, and CHX groups (indicated by red arrows). By contrast, the teeth in the CP-SAP + L group were observed to have the most intact appearance and fewest caries (Fig. 6D).

**Fig. 7:**
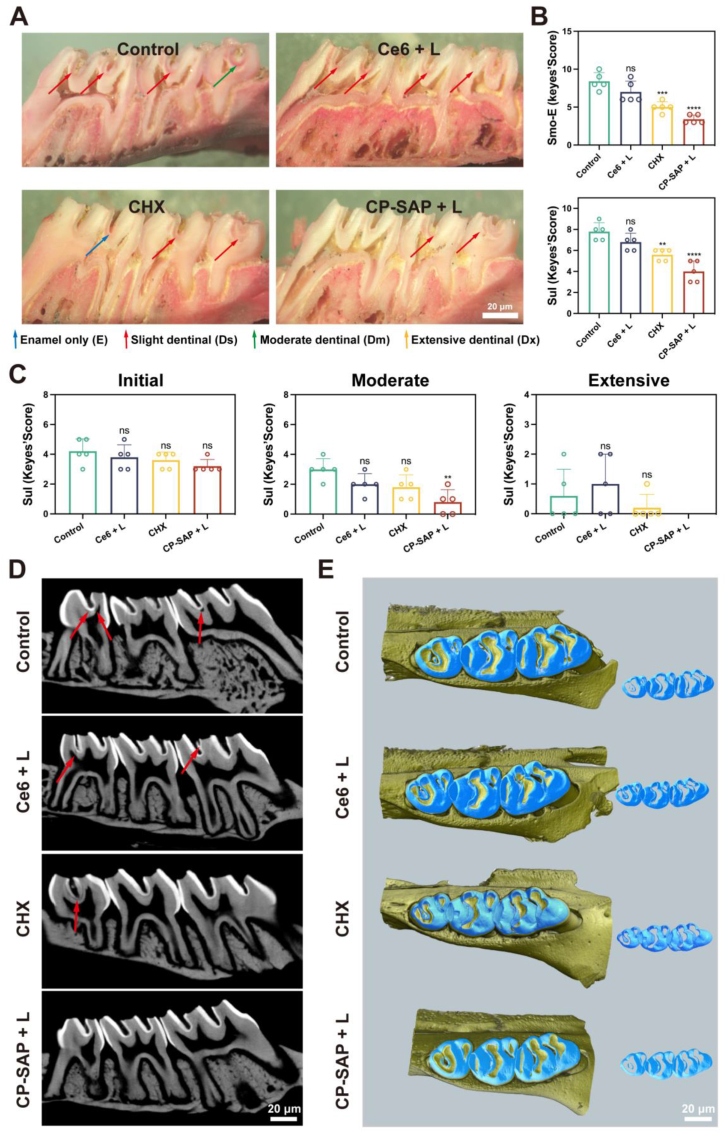
(A) Representative images of caries on sulcal surfaces stained with murexide (blue arrows, affected enamel only, E; red arrows, minimally affected dentin, up to 25 % of the dentin, DS; green arrows, moderate effect on dentin, 25–75 % of the dentin, Dm; yellow arrows, extensive effect on dentin, >75 % of the dentin, Dx). (B, C) Four-level categorization of caries severity on smooth and sulcal surfaces, with total damage including the smooth and sulcal surfaces (E + Ds + Dm + Dx); initial damage to the sulcal surface (Ds + Dm + Dx); moderate damage to the sulcal surface (Dm + Dx); extensive damage to the sulcal surface (Dx). (D) 3D micro-CT sagittal images of maxillary molars (red arrows indicate demineralized areas). (E) 3D reconstructions of micro-CT images of maxillary molars at a certain density threshold, isolating the enamel. Data are presented as mean ± SD, (n = 5), ∗∗p < 0.01, ∗∗∗p < 0.001, ∗∗∗∗p < 0.0001, ns: no significance. Comparisons are between the control and other groups.

To further visualize and observe the therapeutic effects, we stained the tooth samples of each group with murexide. The staining results revealed that the degree of caries in the CP-SAP + L group was significantly lower than that in the other treatment groups (Fig. 7A), and the caries prevention effect was superior to that observed in the CHX group. The Keyes scores corresponding to the smooth and sulcal surfaces of the carious teeth were significantly lower for the CP-SAP + L group than that of the control group (Fig. 7B), especially in terms of the moderate damage on the sulcal surface (p < 0.01) (Fig. 7C). Micro computed tomography (Micro-CT) is a typical oral imaging method used in caries studies of isolated teeth to determine the extent of tooth demineralization [58]. CT images revealed that the CP-SAP + L group had more intact volumes and higher enamel density than the other three groups, which were observed to have more demineralization sites (shaded regions denoted by red arrows) (Fig. 7D). Fig. 7E showed an example 3D image that was stripped and reassembled from a maxillary molar. As can be seen, the CP-SAP + L group had higher enamel mineral density (blue) than those of the other groups.

In conclusion, after 21 days of treatment, CP-SAP + L demonstrated superior efficacy in the prevention and treatment of dental caries in rats compared to traditional CHX gargle. This enhanced effect is attributed to the dual benefits of CP-SAP + L, which not only exhibits antibacterial properties but also promotes sustained remineralization, offering a more comprehensive and long-lasting strategy for caries prevention and treatment. However, the current experimental design has some limitations. Future research should aim to develop animal models or methods that better reflect real clinical conditions, allowing for a more accurate assessment of anti-caries materials. For example, after using antibiotics to remove endogenous bacteria, microbial transplantation could be used to rebuild a microbial ecosystem that more closely resembles what is found in humans. Additionally, gene-editing technologies, like CRISPR, could help target specific microorganisms, offering a more realistic simulation of clinical conditions. Despite these challenges, CP-SAP + L shows strong potential as a comprehensive strategy for preventing dental caries.

### 3.7 Biocompatibility of CP-SAP

**Fig. 8:**
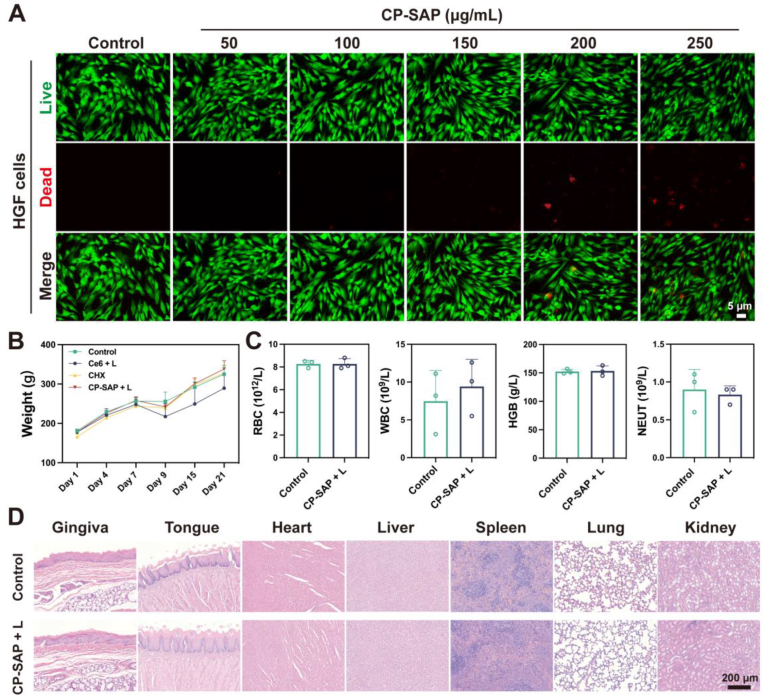
(A) Live/dead staining images of HGF cells treated with different concentrations of CP-SAP. (B) Body weight changes of rat models during the treatment period. (C) Results of routine blood analysis (RBC; WBC; HGB; NEUT) for rats in the control and CP-SAP groups on Day 21. (D) H&E staining of oral gingival mucosa, tongue and other vital organs. Data are presented as mean ± SD, n = 3.

The successful application of CP-SAP in the field of dentistry is dependent on its biocompatibility. To determine the cytotoxicity of CP-SAP, it was initially incubated in vitro with HGF and L929 for 24 h before being subjected to live-dead staining (Fig. 8A, S12). The results showed that in the CP-SAP treatment groups at different concentrations (50–250 μg/mL), the majority of the cells remained viable, with a very low proportion of dead cells. During the in vivo therapy, body weight changes in each rat model were monitored at regular intervals. At the end of the treatment, blood, oral gingival mucosa, tongue, and vital organs were collected for routine blood analysis and hematoxylin-eosin (H&E) staining. Apart from the weight loss observed in rats treated with free Ce6 + L, the weight changes in all other groups were within the expected range (Fig. 8B), indicating high biosafety. The reduced body weights in the free Ce6 group may be attributed to its low biodegradation rate, tendency to aggregate, and potential systemic toxicity [59]. Routine blood analysis and H&E staining revealed no abnormalities at the laser irradiation site, in surrounding tissues, or in major organs following CP-SAP + L treatment. Specifically, key hematological markers, including red blood cell count (RBC), white blood cell count (WBC), hemoglobin (HGB), and neutrophils (NEUT), were all within normal ranges (Fig. 8C). Moreover, no signs of oral mucosal conditions such as erosion, ulcers, or inflammation were observed after CP-SAP + L treatment. H&E staining of the heart, liver, and other vital organs also showed no pathological changes (Fig. 8D). These findings confirm the excellent biocompatibility of CP-SAP and support its potential for clinical applications.

## 4 Conclusion

In this study, we successfully developed an innovative multifunctional gargle for dental caries prevention. Our experimental results demonstrated that this gargle rapidly and firmly adhered to the dental surface, effectively inhibiting the formation of cariogenic bacteria and biofilms within 5 min of laser irradiation. Furthermore, it continued to promote in situ remineralization post-irradiation, restoring the mechanical strength of demineralized enamel. Both in vitro and in vivo experiments demonstrated that the gargle's effectiveness in antibacterial activity, remineralization promotion, and overall caries prevention was comparable to, and potentially surpasses, current strategies such as chlorhexidine and fluoride. However, we acknowledge certain limitations in our study. These include limitations in preclinical research, such as the lack of long-term effect and safety evaluation of CP-SAP, the differences between the oral environments of rats and humans, and an insufficient sample size. Additionally, to improve patient compliance and comfort, the 5-min irradiation time could be shortened by developing more efficient strategies to optimize treatment duration. Addressing these challenges will be a key focus of our future research efforts. Nevertheless, the findings of this study remain significant. The multifunctional delivery carrier we developed, with its wet adhesion and remineralization properties, not only ensures long-lasting therapeutic effects but also introduces a novel approach to the development of materials for oral caries prevention.

## CRediT authorship contribution statement

**Jiayi Shi:** Writing – review & editing, Writing – original draft, Funding acquisition, Formal analysis, Data curation, Conceptualization. **Xuekai Qi:** Software, Methodology, Investigation, Funding acquisition. **Ying Ran:** Validation, Resources, Formal analysis, Data curation. **Qiang Zhou:** Software, Resources, Project administration, Methodology. **Yiqin Ding:** Investigation, Data curation, Conceptualization. **Lujian Li:** Visualization, Investigation, Formal analysis. **Youyun Zeng:** Supervision, Methodology, Conceptualization. **Dongchao Qiu:** Resources, Methodology. **Zhibin Cai:** Writing – review & editing, Supervision, Funding acquisition. **Xiaojun Cai:** Writing – review & editing, Writing – original draft, Supervision, Funding acquisition. **Yihuai Pan:** Writing – review & editing, Writing – original draft, Supervision, Methodology, Funding acquisition.

## Data availability

All relevant data supporting the findings of this study are either included within the article and its Supplementary Information files, or are available upon request from the corresponding author.

## Ethics approval and consent to participate

All research involving animals was conducted in strict accordance with the ethical guidelines and regulations set forth by the institution and the nation. The experimental protocols, which included the use of animals, were reviewed and approved by the Institutional Animal Care and Use Committee (IACUC), adhering to the ethical review procedures for animal experiments as stipulated by Wenzhou Medical University (Wenzhou, China) with the approval number wydw2024-0394.

## Funding

This work was supported by the 10.13039/501100001809National Natural Science Foundation of China (82170950, 82272150), 10.13039/501100018684National Health Commission of the People's Republic of China (WKJ-ZJ-2214), and Wenzhou Basic Scientific Research Project (Grant No. Y20240323).

## Declaration of competing interest

Declaration of competing interest the authors declare no competing interest.
