# Supplementary material for: Saliva-acquired pellicle inspired multifunctional gargle with wet adhesion, photodynamic antimicrobial, and In situ remineralization properties for dental caries prevention
Source: Bioact Mater. 2025 Jan 23;47:212–28. doi: 10.1016/j.bioactmat.2025.01.008 (PMC11790425; doi:10.1016/j.bioactmat.2025.01.008)
Supplement: Multimedia component 1 [file mmc1.docx]

**Supplementary Information**

**Title: "**Saliva-Acquired Pellicle Inspired Multifunctional Gargle with Wet Adhesion, Photodynamic Antimicrobial, and In Situ Remineralization Properties for Dental Caries Prevention".

**Supplementary Figures**

**
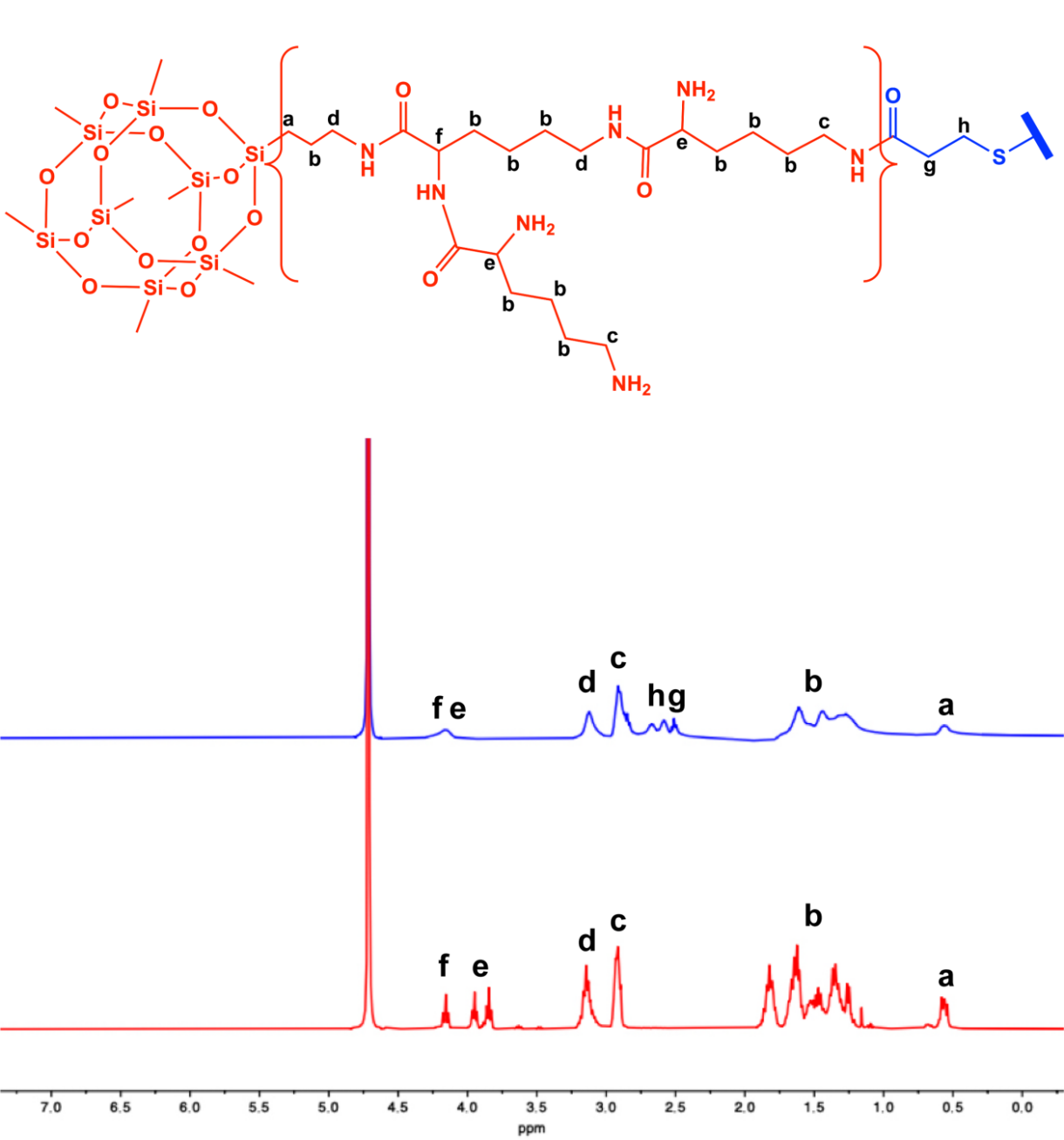
**

**Fig. S1.** ^1^H nuclear magnetic resonance spectra of G2-Lys and PDN in Deuterium oxide (D_2_O).

| m_Ce6_ : m_PDN_ | Size (nm) | PDI | Encapsulation efficiency (%) | loading capacity (%) |
| --- | --- | --- | --- | --- |
| 1:10 | 183.1 | 0.247 | 90.38 | 6.46 |
| 1:15 | 158.5 | 0.200 | 89.42 | 6.35 |
| 1:20 | 146.5 | 0.223 | 87.36 | 6.21 |

**Table. S2.** The encapsulation efficiency and loading capacity of Ce6 in Ce6@PDN (CP).


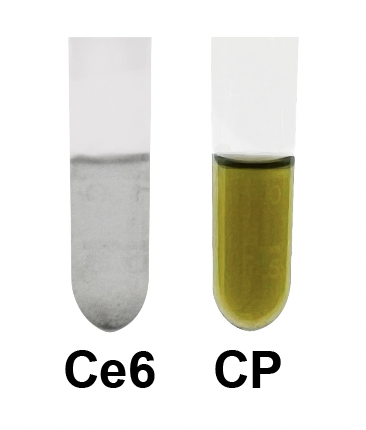


**Fig. S3.** Photographs of free Ce6 aqueous solution and CP aqueous solution.

| m_SAP_ : m_CP_ | Size (nm) | PDI | Graft ratio (%) |
| --- | --- | --- | --- |
| 1:20 | 657.2 | 0.634 | 91.5 |
| 1:30 | 488.5 | 0.302 | 91.8 |
| 1:40 | 240.7 | 0.164 | 91.6 |
| 1:50 | 239.8 | 0.181 | 86.5 |

**Table. S4**. Particle size and graft ratio of CP-SAP prepared at different SAP/CP weight ratios.


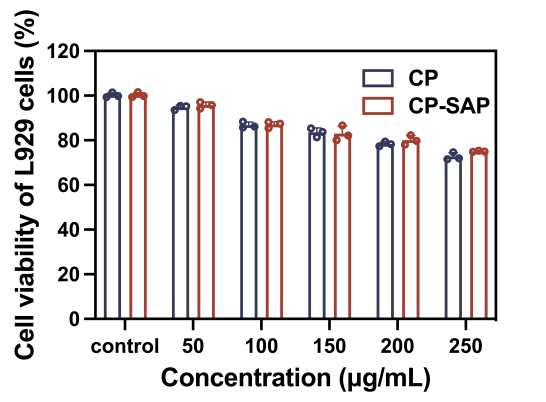

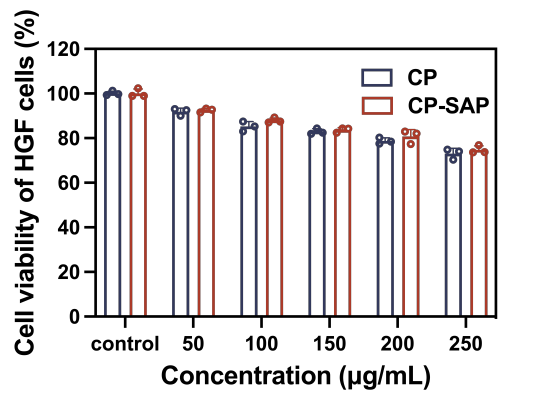


**Fig. S5.** Using the CCK-8 assay to evaluate the cytotoxicity of L929 and HGF cells after treatment with CP-SAP (50-250 µg/mL) for 3 days.


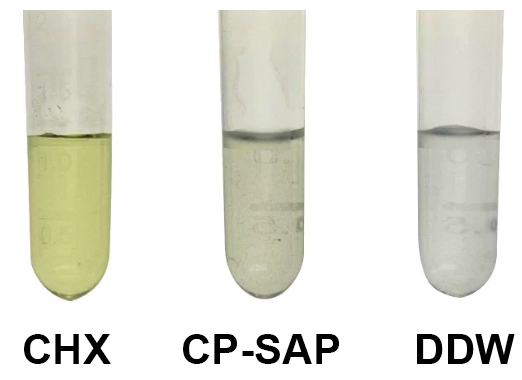


**Fig. S6.** Images of CHX, 200 µg/ml CP-SAP, and DDW.


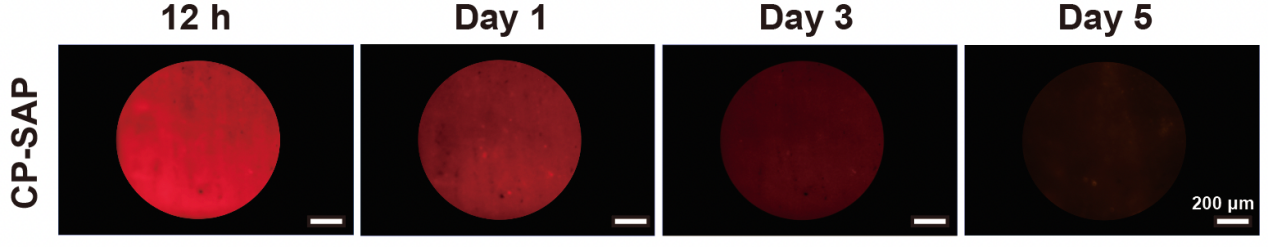


**Fig. S7.** The long-term adhesion capability of CP-SAP in an acidic environment (pH = 5.5).


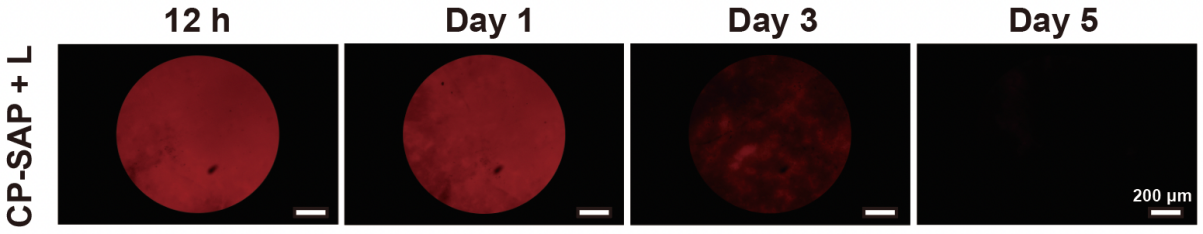


**Fig. S8.** The long-term adhesion capability of CP-SAP + L.


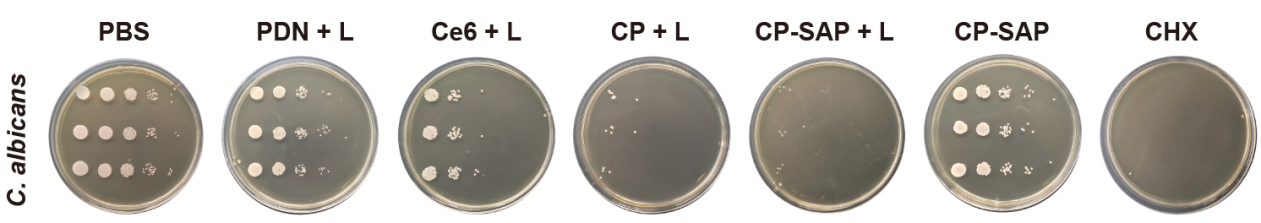


**Fig. S9.** Representative plate images of *C. albicans* after various treatments.

**
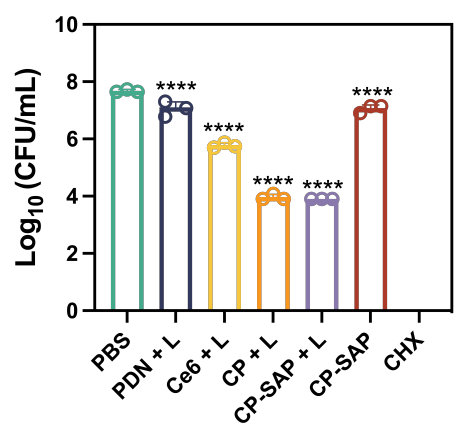
**

**Fig. S10.** The CFU count of *C. albicans* after various treatments.

**
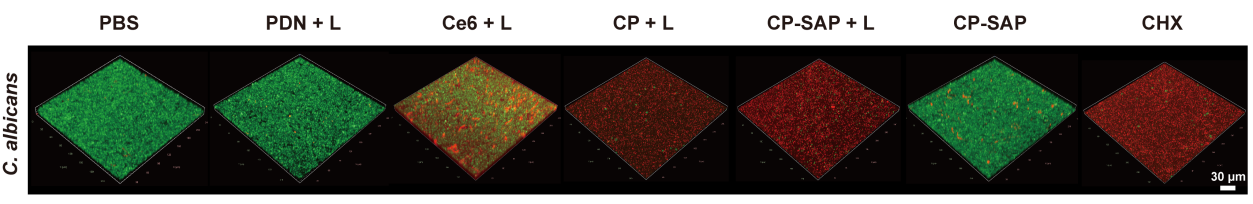
**

**Fig. S11.** Live/Dead staining of *C.albicans* biofilm formation inhibition.


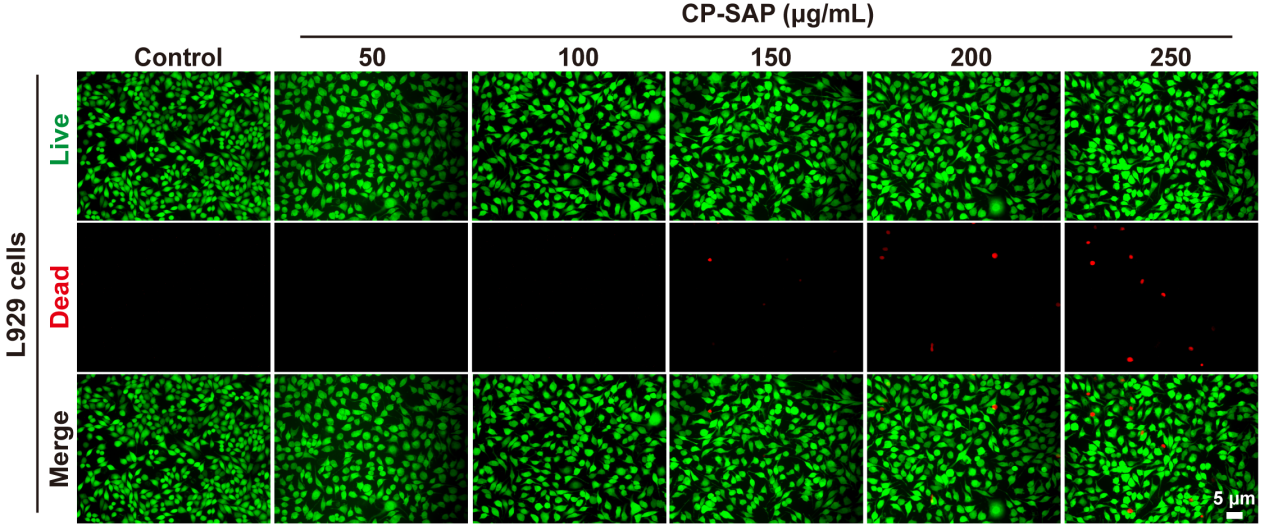


**Fig. S12.** Representative live/dead staining images of L929 cells treated with different concentrations of CP-SAP.
